# Milk yield responses to changes in milking frequency during early lactation are associated with coordinated and persistent changes in mammary gene expression

**DOI:** 10.1186/1471-2164-14-296

**Published:** 2013-05-02

**Authors:** Emma H Wall, Jeffrey P Bond, Thomas B McFadden

**Affiliations:** 1 Department of Animal Science, University of Vermont, Burlington, 05405, USA; 2 Current address: Department of Medicine, University of Vermont, Burlington, VT, 05405, USA; 3 Vermont Genetics Network Bioinformatics Core, University of Vermont, Burlington, 05405, USA; 4 Department of Agricultural, Food and Nutritional Science, University of Alberta, Edmonton, AB, T6G 2P5, Canada; 5 Current address: Division of Animal Sciences, University of Missouri, Columbia, MO, 65211, USA

## Abstract

**Background:**

The lactating mammary gland responds to changes in milking frequency by modulating milk production. This response is locally regulated and, in dairy cows, the udder is particularly sensitive during early lactation. Relative to cows milked twice-daily throughout lactation, those milked four-times-daily for just the first 3 weeks of lactation produce more milk throughout that lactation. We hypothesized that the milk yield response would be associated with increased mammary cell turnover and changes in gene expression during frequent milking and persisting thereafter. Cows were assigned to unilateral frequent milking (UFM; left udder halves milked twice-daily; right udder halves milked four-times daily) on days 1 to 21 of lactation, followed by twice-daily milking for the remainder of lactation. Relative to udder halves milked twice-daily, those milked four-times produced more milk during UFM; the difference in milk yield declined acutely upon cessation of UFM after day 21, but remained significantly elevated thereafter. We obtained mammary biopsies from both udder halves on days 21, 23, and 40 of lactation.

**Results:**

Mammary cell proliferation and apoptosis were not affected by milking frequency. We identified 75 genes that were differentially expressed between paired udder halves on day 21 but exhibited a reversal of differential expression on day 23. Among those genes, we identified four clusters characterized by similar temporal patterns of differential expression. Two clusters (11 genes) were positively correlated with changes in milk yield and were differentially expressed on day 21 of lactation only, indicating involvement in the initial milk yield response. Two other clusters (64 genes) were negatively correlated with changes in milk yield. Twenty-nine of the 75 genes were also differentially expressed on day 40 of lactation.

**Conclusions:**

Changes in milking frequency during early lactation did not alter mammary cell population dynamics, but were associated with coordinated changes in mammary expression of at least 75 genes. Twenty-nine of those genes were differentially expressed 19 days after cessation of treatment, implicating them in the persistent milk yield response. We conclude that we have identified a novel transcriptional signature that may mediate the adaptive response to changes in milking frequency.

## Background

The development and function of many organs is responsive to and regulated by the physiological demands placed upon it [1-3]. The mammary gland is no exception and regular removal of milk is critical to maintaining milk secretion. During early lactation, the milk production potential of the mammary gland is particularly sensitive to the nutritional needs of the offspring such that an increase in demand (milk removal) elicits an increase in milk production [4]. This is an adaptive strategy, regulated at the level of the mammary gland, to meet the demands of the offspring [5]. In dairy production systems, frequent suckling by the offspring can be mimicked by frequent machine milking (3 or more times daily) to stimulate milk production in ruminants by 12 to 14% [6,7]. As a result, frequent milking of dairy cows has become a successful management tool for increasing milk production efficiency and many dairy producers have adopted three times daily milking of their cows.

More recently, it has been reported that increased milking frequency (**IMF**; four times daily milking (**4×**) or more) for the first few weeks during early lactation elicits an increase in milk production that partially carries over through the remainder of that lactation, even after the milking frequency is returned to 2× [4,8-10]. Although the mechanisms underlying this response are unknown, we have shown that both the acute and the long-term increases in milk yield are regulated locally, at the level of the mammary gland [10]. With respect to possible mechanisms for regulating the increase in milk yield in response to IMF, changes in cell number, activity, blood supply and/or mammary metabolism have been proposed [9,11-15].

Recently, Connor et al. [11] investigated the transcriptional response of the mammary gland to IMF during early lactation. They observed changes in mammary expression of genes involved in cell proliferation, cellular remodeling, and nutrient transport. Mammary cell proliferation, however, was not consistently affected [9] and was not affected by IMF in other experiments [12,16]. Therefore, although there is a clear transcriptional response to IMF, the functional implications of this response remain unclear. We recently reported that the milk yield response to IMF is mediated by genes acutely regulated by removal of milk from the mammary gland [15]. Many of the differentially expressed genes have been associated with cell proliferation and apoptosis; however, those processes were not affected by IMF based on direct functional measurements [13]. That experiment was designed to investigate the mechanisms involved in the increase in milk yield early in the response to IMF (day 5 of lactation) and, like other experiments [9,11,12,16], biopsies were obtained during the acute phase of frequent milking treatment, when differential milk yield was increasing. The milk yield response to IMF is dynamic, however, characterized by a marked increase in milk yield during IMF that peaks by day 21 of lactation; followed by a marked, acute decrease in milk yield upon cessation of IMF; and finally a carryover effect that maintains an increased level of milk production that is stable and persistent. Therefore, we adopted a sequential tissue sampling approach to provide insight on the potential mechanisms involved at each of those stages.

In the current experiment, we used a half-udder model and a functional genomics approach to determine the physiological and transcriptional changes associated with each phase of the milk yield response to frequent milking during early lactation. We hypothesized that the dynamic milk yield response would be associated with changes in mammary cell proliferation, apoptosis, and/or gene expression. In addition, we hypothesized that the persistent increase in milk yield would be associated with persistent changes in mammary gene expression. Our objectives were to determine the cellular and transcriptional responses associated with: 1) sustained (21-day) unilateral 4× milking during early lactation; 2) the acute decline in milk yield upon cessation of unilateral 4× milking; and 3) the persistent increment in milk yield after resumption of 2× milking in glands initially milked 4×. Our results revealed a coordinated transcriptional response of at least 75 genes that was strongly associated with differential milk yield over time, implicating those genes in the autocrine regulation of milk production. In addition, 29 of these genes were differentially expressed on day 40 of lactation, suggesting they may regulate the stable and persistent increment in milk yield after 4× milking in early lactation.

## Results

### Milk yield

**Figure 1:**
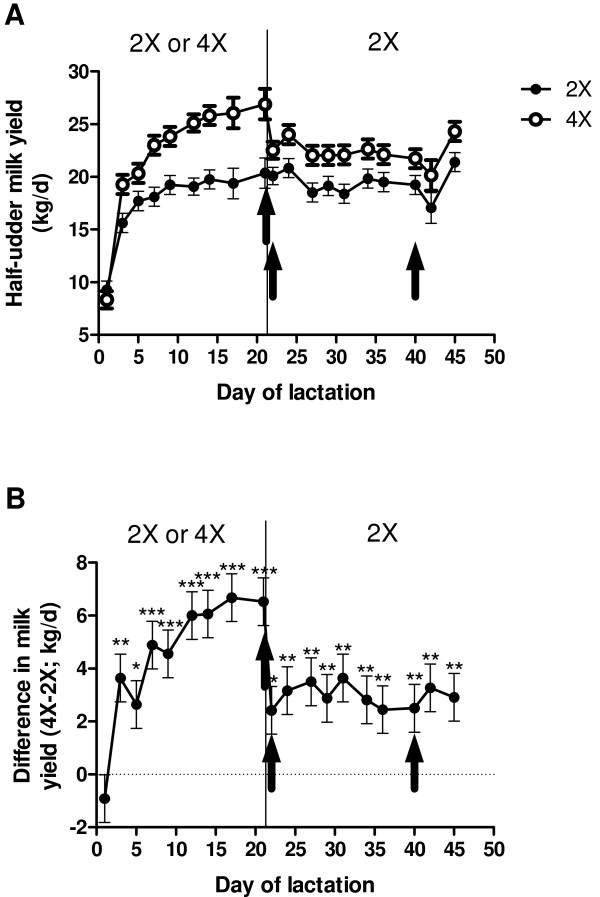
**Changes in milk production in response to IMF.A**. Total milk yield or **B**. difference in milk yield between udder halves, of 6 cows assigned to unilateral frequent milking (UFM; twice daily milking (2×) of the left udder half, four times daily milking (4×) of the right udder half) for days 1 to 21 of lactation. Quartermilk production was measured through 45 d of lactation, and milk production from the 2× udder half was subtracted from that of the 4× udder half to obtain differential yield. There was no difference between udder halves in milk production on day 1 of lactation (*P* > 0.30). Milk production of the 4× udder half increased dramatically (*P* < 0.001) during UFM and was greater than that of the 2× udder half through day 45 of lactation (*P* < 0.01). Black vertical arrows indicate timing of biopsy sampling.

On day 1 of lactation, udder halves produced similar amounts of milk (*P* > 0.30; Figure 1A and B). During UFM, milk production from the 4× udder half increased dramatically and on day 21, average milk production of 2× and 4× udder halves was 20.3 ± 0.8 vs. 26.9 ± 0.8 kg/day, respectively (Figure 1A and B); hence the milk yield differential was 6.6 kg/day. During the entire UFM treatment period, differential milk production of 4× udder halves averaged 5.1 ± 0.6 kg/day more milk than the 2× udder half (*P* < 0.001; Figure 1A and B). Immediately after cessation of UFM, milk production from the 4× udder halves decreased (*P* < 0.01) by day 23; however, the 4× udder half continued to produce 2.7 ± 0.3 kg/day more milk than the 2× udder half through d 180 of lactation (*P* < 0.05; Figure 1A and B; data shown are through day 45 of lactation only).

### Mammary cell proliferation and apoptosis and tissue architecture

**Figure 2:**
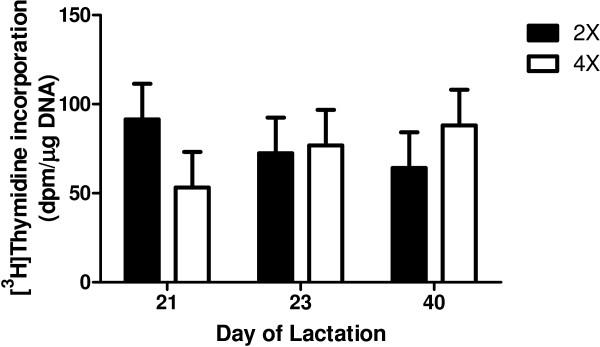
**Incorporation of [**^**3**^**H]-thymidine into DNA of mammary tissue of cows (n = 6) assigned to unilateral frequent milking (UFM; twice daily milking (2×) of the left udder half, four times daily milking (4×) of the right udder half) for days 1 to 21 of lactation.** Mammary biopsies from both udder halves were obtained on d 21, 23, and 40 of lactation. Each bar represents Least Squares mean ± pooled standard error. Incorporation of [^3^H]-thymidine into DNA was not affected by 4× milking or by day of lactation (*P* ≥ 0.80).

**Figure 3:**
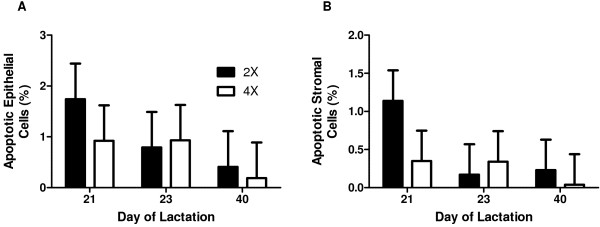
**Mammary cell apoptosis.** Percentage of apoptotic **A**. epithelial or **B**. stromal cells in mammary tissue of cows (n = 6) assigned to unilateral frequent milking (UFM; twice daily milking (2×) of the left udder half, four times daily milking (4×) of the right udder half) for days 1 to 21 of lactation. Mammary biopsies from both udder halves were obtained on d 21, 23, and 40 of lactation. Each bar represents Least Squares mean ± pooled standard error. Mammary cell apoptosis was not affected by 4× milking or by day of lactation (*P* ≥ 0.30).

**Figure 4:**
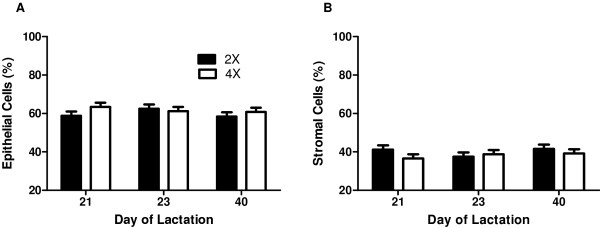
**Mammary cell population dynamics.** Percentage of **A**. epithelial or **B**. stromal cells in mammary tissue of cows (n = 6) assigned to unilateral frequent milking (UFM; twice daily milking (2×) of the left udder half, four times daily milking (4×) of the right udder half) for days 1 to 21 of lactation. Mammary biopsies from both udder halves were obtained on d 21, 23, and 40 of lactation. Each bar represents Least Squares mean ± pooled standard error. The proportion of mammary epithelial and stromal cells was not affected by 4× milking or by day of lactation (*P* ≥ 0.40).

Incorporation of [^3^H]-thymidine into DNA *in vitro* averaged 74 ± 20 disintegrations per min/μg of DNA and was not affected by 4× milking or by day of lactation (*P* ≥ 0.80; Figure 2). The percentage of epithelial cells or stromal cells that were TUNEL positive averaged 0.73 ± 0.70 and 0.38 ± 0.40, respectively, and was not affected by 4× milking or by day of lactation (*P* ≥ 0.30; Figure 3A and B). Proportions of tissue space occupied by epithelial or stromal cells were remarkably consistent and were not affected by treatment or time (*P* ≥ 0.40; Figure 4A and B).

### Microarray analysis of gene expression

#### Identification of genes associated with milk yield

One of the main objectives of our microarray experiment was to identify genes whose expression changed in association with changes in differential milk yield. Therefore, we analyzed the data to identify genes whose differential expression (4× minus 2×) reversed (e.g., changed from positive to negative or vice-versa) from day 21 of lactation (during UFM) to day 23 of lactation (after UFM had ceased). We identified 1637 genes that exhibited a significant reversal or reduction in differential expression (*P* < 0.05). To refine the number of candidates, we then imposed an additional filter to include only those genes whose differential gene expression (4× minus 2×) changed by a magnitude of ≥ 2 between day 21 and day 23 of lactation (75 genes; *P* ≤ 0.05; Table 1). Five of the identified genes were non-annotated transcribed loci with no significant homology to known transcripts (based on homology searches using the Basic Local Alignment Search Tool (**BLAST**)).

**Table 1:** **Mammary expression of genes regulated by changes in milking frequency in dairy cows during early lactation**^**1**^

**Gene Title**	**Gene Symbol**	**Cluster**^**4**^	**Fold change**^**2**^**(d21)**	***P*-value (d21)**	**Fold change (d23)**	***P*-value (d23)**	**Fold change (d40)**	***P*-value (d40)**	**Time**^**3**^**(*P*-value)**	**Probe ID**
Aldehyde dehydrogenase 1 family, member A3	ALDH1A3	1	−1.90	0.003	1.08	0.703	−1.27	0.233	0.016	Bt.8798.1.S1_at
**Apolipoprotein E**	**APOE**	**1**	**−1.73**	**0.024**	**1.18**	**0.480**	**−1.47**	**0.101**	**0.037**	**Bt.5215.1.S1_at**
MHC class I heavy chain	BOLA	1	−1.62	0.018	1.24	0.267	−1.11	0.594	0.016	Bt.29824.1.S1_s_at
Chromosome 20 open reading frame 108 ortholog	C13H20ORF108	1	−1.78	0.001	1.18	0.290	−1.57	0.007	0.002	Bt.1858.1.A1_at
**Complement component 4 binding protein, alpha**	**C4BPA**	**1**	**−1.75**	**0.045**	**1.68**	**0.061**	**−1.32**	**0.308**	**0.008**	**Bt.4707.1.A1_at**
**Chemokine (C-C motif) ligand 2, 11**	**CCL-2, -11**	**1**	**−1.99**	**0.003**	**1.12**	**0.566**	**−1.33**	**0.172**	**0.010**	**Bt.2408.1.S1_s_at**
**Complement factor B**	**CFB**	**1**	**−2.46**	**0.001**	**1.35**	**0.226**	**−1.82**	**0.021**	**0.002**	**Bt.13542.1.S1_at**
**Complement factor I**	**CFI**	**1**	**−3.26**	**0.002**	**1.54**	**0.204**	**−2.16**	**0.029**	**0.002**	**Bt.13387.1.S1_at**
**Chitinase 3-like 1 (cartilage glycoprotein-39)**	**CHI3L1**	**1**	**−3.90**	**0.009**	**2.79**	**0.041**	**−2.26**	**0.098**	**0.002**	**Bt.5238.1.S1_at**
**Claudin 4**	**CLDN4**	**1**	**−1.90**	**< 0.001**	**1.06**	**0.679**	**−1.23**	**0.165**	**0.002**	**Bt.12177.1.S1_at**
**Clusterin**	**CLU**	**1**	**−2.42**	**0.004**	**1.46**	**0.185**	**−1.98**	**0.022**	**0.004**	**Bt.12504.1.S1_at**
**Crystallin, alpha B**	**CRYAB**	**1**	**−1.83**	**0.001**	**1.43**	**0.045**	**−1.42**	**0.047**	**0.001**	**Bt.222.1.S1_at**
Colony stimulating factor 1 receptor	CSF1R	1	−1.83	0.002	1.13	0.484	−1.44	0.048	0.008	Bt.21071.2.A1_at
**Cytochrome b-561**	**CYB561**	**1**	**−1.62**	**0.001**	**1.29**	**0.051**	**−1.32**	**0.036**	**< 0.001**	**Bt.4360.1.S1_at**
**Cytochrome b reductase 1**	**CYBRD1**	**1**	**−2.06**	**0.004**	**1.38**	**0.163**	**−2.07**	**0.003**	**0.003**	**Bt.22055.1.S1_at**
Cytochrome P450, family 11, subfamily A, polypeptide 1	CYP11A1	1	−1.97	0.007	1.30	0.264	−1.50	0.090	0.008	Bt.7190.1.S1_at
**Early growth response 1**	**EGR1**	**1**	**−2.36**	**< 0.001**	**1.53**	**0.055**	**−1.83**	**0.009**	**< 0.001**	**Bt.22265.1.S1_at**
**v-erb-b2 homolog 3**	**ERBB3**	**1**	**−1.76**	**< 0.001**	**1.33**	**0.049**	**−1.54**	**0.005**	**< 0.001**	**Bt.19517.1.S1_at**
**v-fos FBJ murine osteosarcoma viral oncogene homolog**	**FOS**	**1**	**−2.68**	**0.001**	**1.33**	**0.257**	**−1.40**	**0.187**	**0.002**	**Bt.2899.1.S2_at**
Forkhead box Q1	FOXQ1	1	−2.82	0.004	1.34	0.370	−1.76	0.092	0.008	Bt.15658.1.S1_at
**Inositol 1,4,5-triphosphate receptor, type 1**	**ITPR1**	**1**	**−1.80**	**0.007**	**1.14**	**0.511**	**−1.70**	**0.014**	**0.018**	**Bt.23606.1.S1_at**
**Jun oncogene**	**JUN**	**1**	**−2.28**	**0.001**	**1.23**	**0.348**	**−1.11**	**0.620**	**0.003**	**Bt.11159.1.S1_at**
**V-kit Hardy-Zuckerman 4 feline sarcoma viral oncogene homolog**	**KIT**	**1**	**−1.82**	**0.011**	**1.19**	**0.421**	**−1.56**	**0.050**	**0.018**	**Bt.26445.1.A1_at**
**Kruppel-like factor 10**	**KLF10**	**1**	**−2.15**	**0.001**	**1.11**	**0.609**	**−1.40**	**0.117**	**0.007**	**Bt.9527.2.S1_at**
**Kruppel-like factor 11**	**KLF11**	**1**	**−1.89**	**0.001**	**1.08**	**0.644**	**−1.42**	**0.054**	**0.007**	**Bt.16916.3.S1_at**
**Keratin 15**	**KRT15**	**1**	**−1.89**	**0.006**	**1.17**	**0.454**	**−1.55**	**0.046**	**0.013**	**Bt.22801.1.S1_at**
**Keratin 7**	**KRT7**	**1**	**−1.84**	**0.002**	**1.21**	**0.278**	**−1.40**	**0.066**	**0.004**	**Bt.8105.1.S1_at**
**Keratin 8**	**KRT8**	**1**	**−2.17**	**0.002**	**1.32**	**0.209**	**−1.51**	**0.072**	**0.002**	**Bt.23608.1.S1_s_at**
**Lectin, galactoside-binding, soluble**	**LGALS3**	**1**	**−2.12**	**0.002**	**1.22**	**0.361**	**−1.51**	**0.063**	**0.004**	**Bt.1416.1.S1_at**
Hypothetical LOC516522	LOC516522	1	−2.04	0.004	1.20	0.415	−1.95	0.007	0.010	Bt.24918.1.A1_at
Similar to Probable phospholipid-transporting ATPase IIA (ATPase class II type 9A)	LOC516579	1	−1.77	0.003	1.13	0.470	−1.41	0.056	0.009	Bt.21546.1.S1_at
Hypothetical protein LOC785058	LOC785058	1	−1.95	0.007	1.54	0.068	−1.15	0.533	0.002	Bt.9679.1.S1_at
Hypothetical protein LOC786129	LOC786129	1	−1.78	0.005	1.21	0.311	−1.37	0.099	0.007	Bt.685.1.A1_at
Similar to Extracellular proteinase inhibitor	LOC787253	1	−1.68	0.005	1.20	0.277	−1.05	0.750	0.006	Bt.9675.1.S1_at
**Leukotriene A4 hydrolase**	**LTA4H**	**1**	**−1.64**	**0.004**	**1.32**	**0.089**	**−1.30**	**0.110**	**0.002**	**Bt.6143.1.S1_at**
**Lactotransferrin**	**LTF**	**1**	**−1.74**	**0.004**	**1.17**	**0.361**	**−1.44**	**0.045**	**0.008**	**Bt.4802.1.S1_at**
Hypothetical LOC614805	MGC165862	1	−2.33	0.004	1.38	0.228	−1.65	0.066	0.004	Bt.9774.1.S1_a_at
**Melanophilin**	**MLPH**	**1**	**−1.82**	**0.006**	**1.24**	**0.284**	**−1.35**	**0.135**	**0.007**	**Bt.29245.1.A1_at**
AT rich interactive domain 5B	ARID5B	1	−1.68	0.010	1.25	0.242	−1.71	0.008	0.010	Bt.13289.2.S1_at
**Plexin domain containing 2**	**PLXDC2**	**1**	**−1.71**	**0.001**	**1.22**	**0.172**	**−1.35**	**0.045**	**0.001**	**Bt.25703.1.A1_at**
**Protease, serine, 22**	**PRSS22**	**1**	**−1.92**	**0.007**	**1.22**	**0.369**	**−1.24**	**0.326**	**0.012**	**Bt.29351.1.A1_at**
**Protein tyrosine phosphatase, receptor type, D**	**PTPRD**	**1**	**−1.88**	**0.007**	**1.12**	**0.603**	**−1.33**	**0.199**	**0.022**	**Bt.18753.1.A1_at**
**Retinol binding protein 1, cellular**	**RBP1**	**1**	**−2.44**	**0.003**	**1.29**	**0.358**	**−1.70**	**0.060**	**0.006**	**Bt.17810.1.S1_a_at**
Regulator of G-protein signaling 2, 24 kDa	RGS2	1	−1.95	0.003	1.14	0.520	−1.37	0.128	0.009	Bt.10855.1.S1_at
**S100 calcium binding protein A14**	**S100A14**	**1**	**−1.83**	**0.004**	**1.13**	**0.520**	**−1.54**	**0.032**	**0.012**	**Bt.21342.1.S1_at**
**S100 calcium binding protein A2**	**S100A2**	**1**	**−2.11**	**0.007**	**1.35**	**0.246**	**−1.52**	**0.108**	**0.007**	**Bt.5970.1.S1_a_at**
**Serum amyloid A 3**	**SAA3**	**1**	**−2.96**	**0.010**	**2.53**	**0.024**	**−1.56**	**0.259**	**0.001**	**Bt.278.1.S1_at**
**Scinderin**	**SCIN**	**1**	**−1.72**	**0.005**	**1.18**	**0.340**	**−1.54**	**0.022**	**0.009**	**Bt.3980.1.S1_at**
**Shisa homolog 2 (Xenopus laevis)**	**SHISA2**	**1**	**−1.85**	**0.011**	**1.19**	**0.447**	**−1.61**	**0.043**	**0.021**	**Bt.22389.1.S1_at**
**Solute carrier family 1, member 1**	**SLC1A1**	**1**	**−1.54**	**0.010**	**1.36**	**0.058**	**−1.36**	**0.055**	**0.003**	**Bt.23659.1.S1_at**
**Sortilin 1**	**SORT1**	**1**	**−1.62**	**0.002**	**1.25**	**0.113**	**−1.19**	**0.216**	**0.001**	**Bt.23586.1.S1_at**
**Sex determining region Y)-box 4**	**SOX4**	**1**	**−1.89**	**0.007**	**1.23**	**0.342**	**−1.78**	**0.013**	**0.011**	**Bt.12453.1.A1_at**
**Sex determining region Y-box 9**	**SOX9**	**1**	**−1.80**	**0.023**	**1.16**	**0.543**	**−1.84**	**0.019**	**0.042**	**Bt.6983.1.S1_at**
**Tumor-associated calcium signal transducer 2**	**TACSTD2**	**1**	**−2.19**	**0.005**	**1.27**	**0.351**	**−1.43**	**0.166**	**0.009**	**Bt.1694.1.S1_at**
Transcription factor CP2-like 1	TFCP2L1	1	−1.81	0.004	1.16	0.433	−1.42	0.067	0.009	Bt.28282.1.S1_at
**Thrombospondin 1**	**THBS1**	**1**	**−2.11**	**0.001**	**1.15**	**0.467**	**−1.57**	**0.023**	**0.003**	**Bt.5301.1.S1_at**
**Zinc finger protein 36**	**ZNF36**	**1**	**−1.67**	**0.001**	**1.21**	**0.158**	**−1.40**	**0.017**	**0.001**	**Bt.19706.1.S1_at**
Transcribed locus	-	1	−1.84	0.012	1.20	0.428	−2.12	0.003	0.020	Bt.18855.1.A1_at
**Transcribed locus**	**-**	**1**	**−2.13**	**0.007**	**1.37**	**0.230**	**−2.07**	**0.009**	**0.007**	**Bt.13764.1.S1_at**
Transcribed locus	-	1	−1.56	0.003	1.41	0.016	−1.45	0.010	< 0.001	Bt.25328.1.A1_at
**Transcribed locus**	**-**	**1**	**−1.68**	**0.011**	**1.21**	**0.316**	**−1.48**	**0.047**	**0.013**	**Bt.24919.1.S1_at**
**IQ motif containing GTPase activating protein 1**	**IQGAP1**	**2**	**−1.58**	**0.015**	**1.46**	**0.042**	**1.22**	**0.267**	**0.003**	**Bt.21982.1.S1_a_at**
**RNA binding motif protein 5**	**RBM5**	**2**	**−1.68**	**0.005**	**1.46**	**0.032**	**1.17**	**0.358**	**0.001**	**Bt.27798.1.A1_at**
**S100 calcium binding protein A12**	**S100A12**	**2**	**−1.58**	**0.077**	**1.32**	**0.268**	**1.00**	**0.986**	**0.046**	**Bt.357.1.S1_at**
**ATPase type 13A4**	**ATP13A4**	**3**	**1.78**	**0.005**	**−1.13**	**0.517**	**1.04**	**0.835**	**0.015**	**Bt.25459.1.A1_at**
**Frizzled family receptor 5**	**FZD5**	**3**	**1.95**	**0.001**	**−1.06**	**0.769**	**1.20**	**0.335**	**0.011**	**Bt.16100.2.S1_at**
Hypothetical protein LOC540918	LOC540918	3	1.89	0.001	−1.14	0.435	1.21	0.246	0.003	Bt.12714.1.A1_at
**Lipin1**	**LPIN1**	**3**	**1.90**	**0.004**	**−1.09**	**0.661**	**1.18**	**0.424**	**0.017**	**Bt.6642.2.S1_at**
**Myosin binding protein C, slow type**	**MYBPC1**	**3**	**1.78**	**0.006**	**−1.14**	**0.493**	**1.25**	**0.250**	**0.015**	**Bt.23229.1.S1_at**
**Nebulin**	**NEB**	**3**	**1.75**	**0.002**	**−1.21**	**0.238**	**1.28**	**0.134**	**0.003**	**Bt.14153.1.S1_at**
Oxidative stress induced growth inhibitor family member 2	OSGIN2	3	1.73	0.001	−1.18	0.265	1.04	0.771	0.002	Bt.21696.1.S1_at
Transcribed locus	-	3	1.82	0.009	−1.29	0.232	1.03	0.879	0.008	Bt.21137.2.S1_at
**Low density lipoprotein related protein 2**	**LRP2**	**4**	**1.94**	**< 0.001**	**−1.13**	**0.315**	**−1.01**	**0.950**	**< 0.001**	**Bt.8371.1.S1_at**
**Myostatin**	**MSTN**	**4**	**1.78**	**< 0.001**	**−1.19**	**0.188**	**−1.28**	**0.067**	**< 0.001**	**Bt.8967.1.S1_at**
Solute carrier family 34	SLC34A2	4	1.74	0.007	−1.18	0.389	−1.02	0.914	0.013	Bt.21137.1.S1_at

^1^Cows (n = 6) were assigned to twice daily milking (**2×**) of the left udder half and four times daily milking (**4×**) of the right udder half during d 1 to 21 of lactation. Mammary biopsies from both udder halves were obtained on d 21, 23, and 40 of lactation. Differential gene expression was detected using Affymetrix GeneChip® Bovine Genome Arrays.

^2^Signed fold change (4× vs. 2× udder halves).

^3^Time = change in differential gene expression between 2× and 4× udder halves on day 21 vs. that on day 23 (magnitude of difference ≥ 2; *P* ≤ 0.05).

^4^Genes were grouped into four clusters based on the temporal pattern of differential expression.

Genes in bold were consistently differentially expressed in a previous independent microarray experiment [15].

#### Genes differentially expressed during and after UFM

After we identified genes associated with differential milk yield (Table 1), we then went on to investigate the effect of 4× milking on those 75 genes at each individual timepoint. Within the set of 75 genes, expression of 64 genes was lower, whereas expression of 11 genes was higher, in 4× vs. 2× udder halves during UFM (day 21 of lactation; *P* < 0.05; Table 1). After cessation of UFM (day 23 of lactation), expression of 8 genes was higher in 4× vs. 2× udder halves (*P* ≤ 0.05; Table 1). On day 40 of lactation, expression of 29 of the 75 genes was lower in 4× vs. 2× udder halves (*P* < 0.05; Table 1).

### Cluster analysis

**Figure 5:**
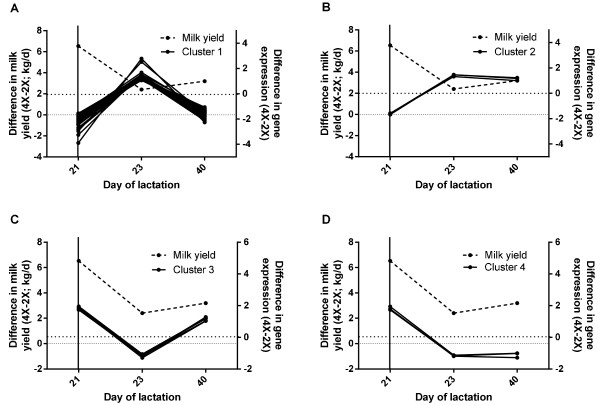
**Temporal pattern of differential gene expression is associated with changes in milk yield.** Cows were assigned to unilateral frequent milking (UFM; twice daily milking (2×) of the left udder half, four times daily milking (4×) of the right udder half) during d 1 to 21 of lactation. Mammary biopsies were obtained from both udder halves on d 21, 23, and 40 of lactation. Gene expression was detected using Affymetrix GeneChip® Bovine Genome Arrays. Genes of which differential expression changed over time were grouped into 4 clusters based on the temporal pattern of differential expression. Cluster 1 (panel **A**; 61genes) included genes whose expression in 4× vs 2× udder halves was lower on day 21, higher on day 23 (after cessation of 4× milking), and subsequently lower on day 40 of lactation. Cluster 2 (panel **B**; 3 genes) included genes whose expression in 4× vs. 2× udder halves was lower on day 21, higher on day 23, and remained higher on day 40 of lactation. Cluster 3 (panel **C**; 8 genes) included genes whose expression in 4× vs. 2× udder halves was higher on day 21, lower on day 23, and higher on day 40. Finally, cluster 4 (panel **D**; 3 genes) included genes whose expression in 4× vs. 2× udder halves was higher on day 21, lower on day 23, and remained lower on day 40. Differential milk yield is plotted on the left Y-axis and differential expression of genes in each cluster is plotted on the right Y-axis. Gene expression data are presented as signed fold change (4× minus 2×) for each gene. Solid vertical line represents cessation of UFM and return of all 4 glands to 2× milking on day 21 of lactation.

Because there were clear differences in the expression of the 75 genes over time, we grouped them into 4 clusters based on the temporal pattern of differential expression (Figure 5 A-D). Cluster 1 (Figure 5A; 61 genes) included genes whose relative expression in 4× vs. 2× udder halves was lower on day 21, higher on day 23 (after cessation of 4× milking), and subsequently lower on day 40 of lactation. Cluster 2 (Figure 5B; 3 genes) included genes whose expression in 4× relative to 2× udder halves was lower on day 21, higher upon cessation of 4× milking on day 23, and remained higher or did not differ on day 40 of lactation. Cluster 3 (Figure 5C; 8 genes) included genes whose expression was higher in 4× vs. 2× udder halves on day 21, lower upon cessation of 4× milking on day 23, and higher or not different in 4× vs. 2× udder halves on day 40. Finally, cluster 4 (Figure 5D; 3 genes) included genes whose expression in 4× relative to 2× udder halves was higher on day 21, lower on day 23 and remained lower or not different on day 40. The average change in differential expression of genes in clusters 1 and 2 was negatively correlated with the change in differential milk yield between day 21 and day 23 of lactation (r = −0.56; *P* ≤ 0.05 for both clusters) as well as across all three time points (r = −0.57; *P* ≤ 0.20 and −0.99 *P* ≤ 0.02 for clusters 1 and 2, respectively; Figure 5A and B; compare solid lines to dashed line), whereas the average differential expression of genes in clusters 3 and 4 was positively associated with differential milk yield between day 21 and day 23 of lactation (r = +0.56; *P* ≤ 0.05 for both clusters) as well as across all three time points (r = +0.64; *P* ≤ 0.10 and +0.99; *P* ≤ 0.20 for clusters 3 and 4, respectively; Figure 5C and D; compare solid black lines to dashed line).

### Pathway analysis

Ingenuity Pathway Analysis (IPA; Ingenuity® Systems, http://www.ingenuity.com) was used to gain insight into functions of, and to detect potential connections between, the 75 genes whose expression was correlated with differential milk yield. Pathway analysis indicated that within the set of 75 genes, there were known interactions among 28 of them (Additional file 1: Figure S1). Expression of five of those genes was measured using RT-qPCR (Additional file 2: Figure S2), and the results for each gene were consistent with the microarray findings for the effect of 4× and day of lactation on mammary gene expression. Not surprisingly, IPA did not provide useful findings based on this small set of 75 genes. Therefore, we constructed four larger sets of differentially expressed genes that were selected based solely on a *P*-value cutoff (*P* < 0.01) for the effect of IMF at each of the three timepoints and for the change in differential expression (4× minus 2×) of genes from day 21 to day 23. Genes that were differentially expressed during IMF, on day 21 of lactation (824; *P* < 0.01), were predicted by IPA to be associated with the inhibition of transforming growth factor (**TGF**)-β and interferon-γ (**IFNG**) signaling in 4×- relative to 2×-udder halves (Additional file 3: Table S1), and were predicted to enrich several canonical pathways, including several growth factor and cytokine signaling pathways, as well as some associated with leukocyte signaling or function (Additional file 4: Table S2). There were no significant IPA findings based on differential gene expression on day 23 of lactation, probably due to the small number of genes (42; *P* < 0.01). Genes that were differentially expressed on day 40 of lactation, 19 days after UFM had ceased (785; *P* < 0.01), were associated with weak activation of insulin-like growth factor (**IGF**)-1 (Additional file 3: Table S1) and significant enrichment of canonical pathways including several in common with those identified for day 21, as well as several unique to day 40, notably including prolactin (**PRL**), growth hormone (**GH**), and IGF-1 signaling (Additional file 4: Table S2). Analysis of genes that exhibited a significant change in differential expression from day 21 of lactation to day 23 (426; *P* < 0.01), predicted differential activation of several cytokines and growth factors including GH, IGF-1, PRL, and TGF-β (Additional file 3: Table S1), as well as significant enrichment of canonical signaling pathways for several growth factors and cytokines (Additional file 4: Table S2). In general, analysis of this latter set of genes did not identify unique regulatory molecules or signaling pathways (most were in common with day 21 and a few with day 40) but often linked different genes to these common responses.

## Discussion

In agreement with our previous observations [10,17], unilateral 4× milking elicited an acute increase in milk yield that partially persisted long after cessation of treatment. Half-udder milk yield was measured once weekly in our previous experiments, whereas it was measured every 3 days in the current experiment. These additional measurements confirmed that the acute milk yield response to 4× was indeed rapid and peaked by day 17. About 60% of the peak difference in milk yield between 4× and 2× udder halves was lost between cessation of 4× on day 21 and the next measurement on day 24. Nevertheless, the difference in yield then stabilized and averaged an increment of 2.5 kg/day for the remainder of lactation. This increment represented about 40% of the peak difference or about a 15% increase relative to milk yield of 2×-udder halves. As expected, repeated biopsy of mammary tissue did not appear to influence the difference in milk yield.

The effect of IMF on mammary cell proliferation and apoptosis has been investigated previously, but interpretation of findings has differed. Hale et al. [9] reported increased mammary cell proliferation (measured by tritiated thymidine incorporation) and increased epithelial cell apoptosis (measured by TUNEL) on day 7 of lactation in cows that were milked 4× for the first 3 wk of lactation, compared to cows milked 2×, and concluded that milk yield responses may result from increased proliferation. However, those cellular responses were not observed in all groups of cows milked 4× and an alternative marker of proliferation, Ki67-expression, did not yield consistent results. Overall, indicators of proliferation or apoptosis were not consistently related to milk yield responses, hence an equally plausible interpretation of their results would be that there was no significant effect on cell population dynamics. Consistent with the latter interpretation, both Norgaard et al. [12] and Wall et al. [13,16] reported that despite eliciting an increase in milk yield, there was no significant effect of IMF on mammary cell apoptosis or proliferation during [13,16] or after [12] IMF treatment. The results of the current study are consistent with those observations. In addition, we found no indication that the milk yield response was related to changes in tissue architecture, based on the similar proportions of epithelial and stromal cells.

We identified a set of 75 genes whose expression was associated with differential milk yield. Many of these genes were previously identified as responsive to IMF in dairy cows [11,15], and the direction of differential gene expression in the current study agreed with previous observations. Moreover, in our previous investigation of the acute response to IMF on day five of lactation [15], we identified 54 of the genes listed in Table 1 (marked in bold) as putative responders to removal of milk from the udder and concluded that they must be among those that mediate the milk yield response to IMF. Our current results support that conclusion, and reveal that many genes previously identified as responders to 5 days of IMF [15] were not only also differentially expressed after 21 days of IMF, but their differential expression on day 21 was reversed on day 23, concomitant with a decline in differential milk yield. These two lines of evidence both link differential expression of these genes to changes in milking frequency and the resultant milk yield responses, suggesting that they are coordinately regulated and may comprise a novel mechanism for autocrine regulation of milk yield and mammary adaptation to IMF [5].

It was notable that of the 75 genes associated with differential milk yield, 64 were down-regulated in response to 4× milking whereas only 11 were up-regulated. Down-regulation of gene expression in response to functional stimulation of the mammary gland has been observed previously [11,18,19]. In agreement with our observations, Connor et al. [11] reported that the majority of differentially expressed genes were down-regulated in response to 4× milking. During the transition from late pregnancy to lactation in both mice [19] and cows [18] most of the differentially expressed genes in the mammary gland are down-regulated. In contrast, during mammary involution and loss of secretory function, more genes are up-regulated than are down-regulated [19]. Lemay et al. [19] speculated that pro-lactational stimulus of the mammary gland is not associated with gain of function, but is associated with the suppression of broad functions thereby sparing metabolic support for the specific function of milk synthesis. The stimulus of increased milking frequency may elicit additional streamlining of cellular functions, akin to homeorhetic support of lactation.

One of our objectives was to identify the genes and functions involved in each phase of the milk yield response to IMF; those phases being the acute increase in milk yield during IMF; the partial decrease in milk yield after cessation of IMF, and the persistent increment in milk yield that remained long after IMF. Based on the results of IPA analysis, it was clear that there were both common and distinct pathways responsive during vs. after IMF. For example, differential activation of IGF-1 was predicted by IPA both during and after IMF, whereas inhibition of complement component 5 was predicted during IMF only. Pathway analysis also predicted that at least nine cytokines or growth factors were in an inhibited state during IMF but it is not clear whether or how this may relate to the milk yield response or mammary function. The second phase of the response involved the reduction of difference in milk yield between udder halves after cessation of 4× milking. Despite this dynamic change, IPA did not predict effects on any canonical pathways or regulatory molecules, probably due to the small number of genes that were differentially expressed on day 23 (i.e., most of the genes that were differentially expressed on day 21 returned toward basal levels on day 23 and thus did not differ significantly between udder halves). This result suggests that the decrement in milk yield was associated with reduction of pro-lactational gene expression rather than activation of novel, inhibitory genes. This reasoning is supported by the finding that most genes whose expression was correlated with the reduction in differential milk yield from day 21 to 23 were predicted to be associated with signaling of multiple growth factors and cytokines. With respect to the third, or carryover, phase of the milk yield response (day 40 of lactation), IPA predicted weak activation of IGF-1 signaling and enrichment of numerous canonical signaling pathways, including GH, IGF-1, and PRL, all of which have been linked to regulation of mammary development and cell activity [20-22]. In addition, both IGF-1 and PRL and have been hypothesized to be involved in the response of the mammary gland to IMF [13,16,23,24]. Therefore, although IPA did not provide insight into functional significance of differentially expressed genes, it did predict changes in signaling molecules and pathways that may mediate IMF-induced changes in milk yield and mammary function. Notably, those pathways were predicted to remain affected long after cessation of treatment, perhaps maintaining the sustained increment in milk production.

To gain additional insight into adaptation of the mammary gland to altered milking frequency, we used evidence from the literature, as well as The Gene Ontology (http://www.geneontology.org), to determine functions associated with genes differentially expressed in response to IMF that might regulate milk yield responses.

Of the differentially expressed genes that were downregulated during 4× and were negatively correlated with milk yield, several have been previously linked to mammary development, differentiation and remodeling; all of which may be functionally relevant to the adaptations of the mammary gland to changes in milking frequency. Chitinase 3-like (**CHI3L**)-1 is a secreted protein that is associated with mammary involution and remodeling in dairy cows [25,26], goats [27,28], and sheep [29]. More recently, expression of *CHI3L1* mRNA was linked to lactogenesis of the mammary gland in cell culture [22]. In other tissues, *CHI3L1* is involved in cellular differentiation and remodeling [30-32], and also in the cellular response to mechanical stimuli and pressure [33-35]. It is therefore not surprising that our results have consistently shown that *CHI3L1* is involved in the response of the mammary gland to the stimulus of IMF. Similar to *CHI3L1*, clusterin is a secreted protein that has been associated with involution and remodeling of the mammary gland [36-38]. In addition, clusterin has been used as a marker for apoptosis and DNA damage in both mammary gland and prostate [39-41]. Consistent with a role in the response of the mammary gland to IMF in the current experiment, mammary expression of clusterin is down-regulated by milk removal in rats [36]. Keratin (**KRT**)-8 expression was also lower in 4× vs. 2× udder halves, consistent with previous observations [15]. Conversely, expression of *KRT8* was increased during decreased milking frequency [42]. It has been reported that *KRT8* can protect liver cells from Fas-mediated apoptosis by preventing DNA fragmentation [43]. Although we did not detect an effect of 4× milking on mammary cell apoptosis, it is possible that IMF rescues “surplus” cells that may otherwise be destined for deletion via apoptosis and instead recruits them into an active state. We have previously discussed this possibility in terms of a “use it or lose it” concept [4]. In support of this idea, sex determining region Y-box (**SOX**)-4 and −9 were down-regulated by UFM, and these genes have been implicated in the determination of cell fate and cellular differentiation during key phases of development in reproductive tissues [44-46]. Therefore, the findings of our manual literature searches complement our IPA results by indicating possible functional roles of individual genes in the adaptive response to IMF that may be associated with long-term changes in mammary cell activity and differential milk yield. Shorten et al. [47] presented a hypothetical model proposing that IMF increases the number of active alveoli by increasing the proportion of active to quiescent cells in the mammary gland, which could increase the number of actively secreting alveoli and enhance milk producing capacity for the remainder of lactation.

Expression of early growth response (**EGR**)-1 and thrombospondin (**THBS**)-1 genes was lower in 4× vs. 2× udder halves both during UFM (day 5 [15] and day 21) and after the carryover increment in milk yield had been established (day 40 of lactation). Early growth response-1 is a mechanosensitive transcription factor that is regulated by physical stress in the vasculature [48] and lung [49], among other tissues. Similarly, *THBS1* plays a role in vascular [50-52] and lung [53] development, and it is often co-regulated with *EGR1*[50,54]. Therefore, the lower expression of these genes in 4× glands might be related to reduced mammary distension in the 4× udder half, due to more frequent bouts of milk removal. Similar transcriptional responses to distension and stretch have been described in bladder [55,56]. In the current model, however, it is unclear how such a mechanical response could continue to influence mammary function even after cessation of IMF.

Several genes were more highly expressed in 4× vs. 2× during UFM and post-treatment when the milk yield increment persisted. This group included lipin-1, low density lipoprotein-related protein (**LRP**)-2, and myostatin (**MSTN**). Low density lipoprotein-related protein-2 is a nutrient transporter involved in the endocytic uptake of vitamin D by mammary tissue [57], and it has been suggested to play roles in mammary cell proliferation and differentiation [58,59]. We have shown that expression of *LRP2* in mammary tissue of dairy cows is physiologically regulated [60], further supporting a specific role for this gene in mammary development and function. Expression of *MSTN*, which is also known as growth and differentiation factor-8, has previously been associated with differentiation of the mammary gland in sows [61] although the opposite has been reported in mice [62]. The current findings support a new role for these genes in the response of the mammary gland to changes in milking frequency and consequent changes in milk yield. Because many genes were differentially expressed both during UFM (days 5 [15] and 21 [present study] of lactation), and during the carryover effect on milk yield (day 40 of lactation), it is plausible that the stimulus of IMF elicited epigenetic programming of the mammary gland.

Epigenetic programming is the process by which cells retain a biological memory of environmental events that occur during critical periods of development. Such events initiate stable changes in gene expression without changing nucleotide sequences [63]. The primary mechanisms regulating epigenetic alterations in gene expression are methylation of cytosine residues on the DNA, and histone modification [63,64]. Although epigenetic programming was initially thought to be stable and heritable, it is now considered to include transient, reversible changes stimulated by environmental conditions [65]. During critical phases of development, epigenetic programming can be initiated by a variety of environmental stimuli, including exposure to hormones, or activity of/demand on the tissue or organ [66]. In the mammary gland, gene expression and cellular function can be controlled by epigenetic mechanisms; Plachot and Lelievre [67] reported that DNA methylation plays a role in mammary cell proliferation and differentiation. Exposure of the virgin rat to the hormones of pregnancy is associated with persistent changes in mammary gene expression, which may be involved in the protective effect of pregnancy against breast cancer [68]. In addition, it has been proposed that epigenetic mechanisms underlie acute changes in mammary function and gene expression in dairy cows [69]. Importantly, some of the genes implicated in the acute (day 5 and 21) and carry-over (day 40) effects of IMF on milk yield are known to be epigenetically regulated including *EGR1*[70], and *THBS1*[71]. Therefore, it is possible that, during early lactation, those genes and perhaps others are susceptible to epigenetic modifications that influence the functional capacity of the mammary gland for the remainder of lactation.

Taken together, the results of this experiment indicate that the transcriptional response to changes in milking frequency is characterized by a dynamic yet coordinated pattern of differential expression of at least 75 genes that was correlated with both up- and down-regulation of milk yield in paired udder halves. Therefore, these genes appear to comprise a novel transcriptional program involved in the autocrine regulation of milk production in the mammary gland. Moreover, based on the persistently altered expression of 29 of these genes, we propose that IMF triggers an epigenetic mechanism that sets a new threshold of expression of those genes and their respective functions as an adaptive strategy for matching mammary output to IMF or, in the wild, to demands of suckling neonates during early lactation. We suggest that this represents a novel form of epigenetic regulation that could be called “lactational programming,” since the stimulus of IMF for a short period during early lactation elicits changes in gene expression and cellular functions that persist long after treatment has ceased.

## Conclusions

We conclude that IMF during early lactation does not alter mammary cell population dynamics but is associated with coordinated changes in expression of 75 genes in the mammary gland. The temporal pattern of differential expression of these genes is correlated with changes in differential milk yield. Moreover, twenty nine of these genes were persistently differentially expressed, further implicating them in the long-term alteration of mammary function. Together, our findings indicate that we may have identified a novel form of epigenetic regulation, which could be termed “lactational programming,” characterized by coordinated and persistent changes in gene expression during and after increased milking frequency. Those genes which remain differentially expressed may mediate the persistent effect on milk yield resulting from increased milking frequency during early lactation of dairy cows.

## Methods

### Animals and treatments

Six multiparous Holstein cows were assigned at parturition to unilateral frequent milking (**UFM**; twice daily milking (**2×**) of the left udder half, four-times daily milking (**4×**) of the right udder half) during days 1 to 21 of lactation. Thereafter, both udder halves were milked 2×. Regular milkings took place at 0230 h and 1430 h, and the two extra milkings (during which only the right udder half was milked) took place at 0530 h and 1730 h. The University of Vermont Institutional Animal Care and Use Committee approved animal use and associated procedures.

### Half-udder milking

To quantify the response to UFM, half-udder milk yields were measured using a portable milking system with a milking claw designed to collect milk from each quarter into individual vessels, as previously described [10]. At the first milking post-calving, half-udder milk yields were measured to verify that udder halves produced similar amounts of milk prior to treatment (cows having udders that were unbalanced by >0.7 kg were rejected from the study). Half-udder milk yields were measured during the afternoon milking (1430 h) every 3 ± 1 d until day 45 of lactation. Thereafter, half-udder milking was performed once on days 90, 180 and 270 (±5 d) of lactation. Half-udder milk yields were doubled to estimate daily milk produced by each udder half, which is reported in Figure 1. Data presented are through day 45 of lactation only, as full-lactation results from our previous experiments have been reported elsewhere [10,17].

### Mammary biopsy

Mammary tissue from the midpoint of each quarter was obtained by biopsy using a Pro-Mag Ultra automatic biopsy instrument (Medical Device Technologies, Gainesville, FL) on days 21 (rear quarters), 23 (fore quarters) and 40 (rear quarters) of lactation, as described by Wall et al. [13]. Biopsies were performed at 0500 h, 2.5 h after both udder halves had last been milked. Briefly, prior to mammary biopsy, cows were administered an intravenous injection of 0.50 mL of 20 mg/mL xylazine (0.01 mg/kg BW; Phoenix Pharmaceuticals, St. Joseph, MO) for general sedation. In addition, lidocaine (3 mL; 0.07 mg/kg BW) was administered in a line-block directly above the incision site just prior to biopsy. Biopsy samples of ~15 to 20 mg were obtained and biopsy of the same site was repeated (~3 times) until ~60 mg of tissue was obtained. Biopsy samples were trimmed of extra-parenchymal tissue and a portion (~55 mg) was immediately frozen in liquid nitrogen for subsequent isolation of RNA. The remaining parenchyma (~5 mg) was diced into pieces that were fixed in 10% buffered formalin for subsequent histological analysis.

### Mammary proliferation assay

Mammary parenchyma was diced into explants and approximately 10 mg was incubated in a shaking water bath for 1 h at 37°C in 3 mL of medium 199 (Sigma, St. Louis, MO) supplemented with 1 μCi/mL of [^3^H]-thymidine (33 Ci/mmol, ICN, Irvine, CA) to determine incorporation of [^3^H]-thymidine into DNA. After incubation, explants were blotted, weighed, and frozen in liquid nitrogen. Incorporation of [^3^H]-thymidine into DNA was determined as described by Wall et al. [72], but modified for 10 mg of tissue [13].

### DNA assay

Total DNA in tissue homogenates was measured as described by Labarca and Paigen [73], but modified for assay in a 96-well plate as described previously [72]. Briefly, duplicate 2-μL aliquots of homogenate were pipetted into wells, and 98 μL of DABS-E (0.5 M dibasic NaPO_4_, 0.5 M monobasic NaPO_4_, 2 M NaCl and 0.1 M EDTA) buffer and 100 μL of 2 μg/mL Hoechst 33528 dye (Sigma) were added. Fluorescence was determined using a fluorimeter spectrophotometer (KC4, Bio-Tek Instruments, Winooski, VT). The DNA concentration of homogenates was determined by comparison with a standard curve made by serially diluting calf thymus DNA (Sigma) and was used to calculate the total amount of DNA in the original homogenate.

### Terminal deoxynucleotidyl transferase dUTP nick end labeling (TUNEL)

Detection of apoptotic cells *in situ* was performed utilizing TUNEL, as described by Wall et al. [72]. Briefly, fixed explants were embedded in paraffin, sectioned at approximately 4-μm thickness and mounted onto silanized slides. A commercial TUNEL kit (ApopTag Plus Peroxidase, Chemicon International, Temecula, CA) was used in accordance with the manufacturer’s protocol. After the labeling assay, coverslips were mounted with Cytoseal.

### Quantification of apoptotic cells

Tissue sections were viewed by light microscopy with an Olympus BX41 light microscope (Olympus America Inc., Melville, NY) to quantify labeled cells. Details are described in Wall et al. [72]. Cells were classified as epithelial, stromal, labeled epithelial or labeled stromal cells. All cells in one randomly selected field were counted (at least 2,000 cells) and labeled brown nuclei were readily visible. A cell was classified as labeled when the nuclear staining was at least twice as intense as the background. Slides were coded for counting to prevent observer bias.

### Quantitative histology

Fresh explants of biopsy tissue were fixed in 10% buffered formalin overnight and stored in 70% ethanol. Samples were embedded in paraffin, sectioned at approximately 4 μm thickness and mounted onto silanized slides, with 2 sections mounted per slide. For quantitative histology, slides were stained with hematoxylin and eosin (**H & E**) to allow visualization and characterization of cell types. Briefly, slides were deparaffinized with several changes of xylene, then hydrated by immersion in successive ethanol/water solutions of decreasing ethanol concentration, starting with absolute ethanol and ending with water. The hydrated tissue sections were then stained with H & E, washed with water, and coverslips were mounted with Cytoseal.

Tissue sections were viewed by light microscopy with an Olympus BX41 light microscope to quantify cells. For each sample, 1 microscopic field was quantified. Fields were randomly selected and micrographs were taken at 40× magnification using Optronics Magnafire-SP (W. Nuhsbaum Inc., McHenry, IL). All cells in each field were counted (at least 1500 cells) using Image Pro Express (Media Cybernetics, L.P, Silver Spring, MD). Cells were counted as epithelial if they comprised the epithelial lining, including ductal and alveolar epithelial and myoepithelial cells, and possibly leukocytes if they were present in the alveolar lumen. All cells located in the interstitium were classified as stromal cells, including primarily fibroblasts, adipocytes, leukocytes, and endothelial cells.

### RNA isolation

Total RNA was isolated from each biopsy sample using Trizol reagent (Invitrogen, Carlsbad, CA) according to the manufacturer’s instructions. The RNA was purified using the RNeasy Mini Kit (Qiagen, Valencia, CA) according to the manufacturer’s protocol. Purified RNA was quantified using a Nanodrop ND1000™ spectrophotometer (Thermo Scientific, Wilmington, DE) and quality was assessed using an Agilent 2100 bioanalyzer (Agilent Technologies, Palo Alto, California). The RNA integrity number of all samples was greater than 8. Additional details on RNA processing can be found in Wall et al. [72].

### Affymetrix GeneChip® bovine genome arrays

#### Microarray analysis

RNA amplification and microarray analysis was performed at the University of Vermont microarray core facility using previously described protocols [74]. Briefly, 2 μg of total RNA from each tissue sample were reverse transcribed to the single-stranded cDNA using T7-oligo(dT) primer. T4 DNA polymerase was used to synthesize double-stranded cDNA, which served as a template for *in vitro* transcription using T7 RNA polymerase to produce biotinylated cRNA. The biotinylated cRNAs were fragmented into 50- to 200-base fragments and then hybridized to GeneChip Bovine Genome Arrays for 16 h at 45°C in a rotating Affymetrix GeneChip Hybridization Oven 320. After hybridization, arrays were washed and stained with streptavidin-phycoerythrin on an automated Affymetrix GeneChip Fluidic Station F450. The arrays were scanned with an Affymetrix GeneChip Scanner 2700 and the images quantified using Affymetrix GeneChip Operating Software.

#### Data analysis

The signal intensity for each probe on each chip was calculated from scanned images using GeneChip Operating Software (Affymetrix), and signal intensities were analyzed using BioConductor (http://www.bioconductor.org). Probe intensities were background corrected, normalized, and summarized using the Robust Multichip Average method described by Speed and coworkers [75,76]. An alternative normalization method based on reference genes did not significantly change the results. All microarray data were deposited into NCBI’s Gene Expression Omnibus (accession number GSE27851). To identify genes associated with the decrease in milk yield that occurred after cessation of IMF, the change in differential gene expression from day 21 to 23 of lactation was determined. Only the genes for which differential expression (i.e., the difference between 2× and 4× udder halves) changed from days 21 to 23 of lactation (magnitude of difference ≥ 2; *P* < 0.05) are reported herein.

Evidence from the literature, as well as the Gene Ontology Database, was used to indicate the functions associated with differential gene expression in response to IMF that therefore might regulate the observed milk yield responses.

#### Cluster analysis

Genes of which differential expression changed between day 21 and day 23 of lactation were grouped into four clusters based on the temporal pattern of differential expression. Cluster 1 included genes whose expression in 4×- relative to 2×-glands was lower on day 21, higher on day 23 (after cessation of 4× milking), and subsequently lower on day 40 of lactation. Cluster 2 included genes whose expression in 4×- relative to 2×-glands was lower on day 21, higher on day 23, and remained higher on day 40 of lactation. Cluster 3 included genes whose expression in 4×- relative to 2×-glands was higher on day 21, lower on day 23, and higher on day 40. Finally, cluster 4 included genes whose expression in 4×- relative to 2×-glands was higher on day 21, lower on day 23, and remained lower on day 40.

#### Pathway analysis

Ingenuity Pathway Analysis (**IPA**; Ingenuity® Systems, http://www.ingenuity.com) was used to determine the functions enriched by IMF. The Affymetrix Bovine Array was used as a reference for the analysis, and all other options used were default settings in IPA. To obtain sufficient genes to get meaningful IPA results, microarray data were re-filtered using a threshold of *P* < 0.01 for differential expression of genes at each time point and for the change in gene differential expression (4× minus 2×) on day 21 vs. that on day 23.

### Real-time quantitative PCR

Relative mRNA expression profiles were determined by real-time quantitative PCR (**qPCR**) using a PE 7700 thermal cycler (Applied Biosystems, Foster City, CA). Additional details on PCR can be found in Wall et al. [72]. Primers were designed for bovine *CHI3L1*, clusterin, *EGR1*, *LRP2*, and *SOX4* using Primer 3 software (http://frodo.wi.mit.edu/). Primer sequences for *CHI3L1*, *LRP2*, and *SOX4* can be found in Wall et al. [15]. For clusterin, primer sequences were forward: 5′-caggagatcctggaa-3′ reverse: 5′-actccttccccatcacagtg-3′. For *EGR1*, primer sequences were forward: 5′- aaaagccaagcaaaccaatg -3′ reverse: 5′- tcacacaaaaggcaccaaga -3′. After primer design, the predicted product was BLAST searched against the bovine database to ensure specificity of the primers. All gene expression values were normalized to that of ß-2 microglobulin, which was stable over time and across udder halves.

### Statistical analyses

A one-tailed paired *t*-test (SAS Inst. Inc., Cary, NC; version 9.1) was used to determine the significance of 4× milking on milk production. A two-tailed paired *t*-test (SAS) was used to determine the significance of 4× milking on mammary cell proliferation, apoptosis, percentage of epithelial and stromal cells in the mammary gland, and gene expression measured by RT-qPCR. The general linear models procedure (**GLM**; SAS) was used to determine the significance of time on mammary cell proliferation, apoptosis, percentage of epithelial and stromal cells in the mammary gland, and gene expression measured by RT-qPCR. The Proc Corr procedure (SAS) was used to calculate Pearson’s correlation coefficients and determine significance of associations between differential milk yield per cow and average differential gene expression over time. Correlations were first determined for the changes between day 21 and day 23 of lactation, and then for day 21 through day 40 of lactation.

## Abbreviations

2×: Twice daily milking; 4×: Four-times daily milking; UFM: Unilateral frequent milking; IMF: Increased milking frequency; IPA: Ingenuity pathway analysis; TGF-β: Transforming growth factor beta; IGF-1: Insulin-like growth factor I; PRL: Prolactin; GH: Growth hormone; CHI3L1: Chitinase 3-like-1; KRT8: Keratin-8; EGR1: Early growth response-1; THBS1: Thrombospondin −1; LRP2: Low density lipoprotein-related protein-2; MSTN: Myostatin; H & E: Hematoxylin and eosin; GLM: General linear models

## Competing interests

The authors declare that they have no competing interests.

## Authors’ contributions

EHW carried out the animal trial and microarray experiment, performed the statistical analyses of all data except for the microarray experiment, and drafted the manuscript. JPB participated in the design of the microarray experiment and performed the associated statistical analysis. TBM conceived of the study, participated in its design, and helped to draft the manuscript. All authors read and approved the final manuscript.

## Supplementary Material

Additional file 1: Figure S1 Known potential connections between genes identified by a significant treatment by time interaction, characterized by changes in differential gene expression between 2× and 4× udder halves over time. Figure generated using Ingenuity Pathway Analysis, Ingenuity® Systems (Green = expression decreased, red = expression increased in 4× relative to 2× udder halves on day 21 of lactation). Click here for file

Additional file 2: Figure S2 Expression of A. chitinase 3-like (CHI3L)-1, B. clusterin, C. early growth response (EGR)-1, D. sex determining region Y-box (SOX)-4, and E. low density lipoprotein related protein (LRP)-2 mRNA in mammary tissue of cows assigned to unilateral frequent milking (twice daily milking (2×) of the left udder half, four times daily milking (4×) of the right udder half) during d 1 to 21 of lactation. Mammary biopsies from both udder halves were obtained on d 21, 23, and 40 of lactation. Each bar represents Least Squares Mean ± pooled standard error mRNA expression normalized to β-2 microglobulin. Line graph (secondary x-axis) represents least squares mean difference (4× minus 2×) ± pooled SE mRNA expression normalized to β-2 microglobulin. Solid horizontal line represents zero on the secondary x-axis, indicating no differential expression. Expression of *CHI3L1*, clusterin, *EGR1*, and *SOX4* was decreased in 4× udder halves at 21 days in milk (*P* < 0.07), whereas expression of *LRP2* was increased in 4× udder halves (*P* < 0.01). Expression of *EGR1* was increased in 4× udder halves at 23 days in milk (*P* < 0.10), whereas expression of all other genes was similar across udder halves (*P* > 0.20). Expression of clusterin, *EGR1*, and *SOX4* was decreased in 4× udder halves at 40 days in milk (*P* < 0.07), whereas expression of all other genes was similar across udder halves (*P* > 0.20). Differential expression of all genes changed over time (*P* < 0.07). Click here for file

Additional file 3: Table S1 Predicted activation state of growth factors and cytokines based on differential gene expression induced by IMF^1^. Click here for file

Additional file 4: Table S2 Enrichment of canonical pathways based on genes differentially expressed in response to IMF^1^. Click here for file
