# Supplementary material for: Milk yield responses to changes in milking frequency during early lactation are associated with coordinated and persistent changes in mammary gene expression
Source: BMC Genomics. 2013 May 2;14:296. doi: 10.1186/1471-2164-14-296 (PMC3658990; doi:10.1186/1471-2164-14-296)

Supplemental Figure 1. Known potential connections between genes identified by a significant treatment by time interaction, characterized by changes in differential gene expression between 2X and 4X udder halves over time. Figure generated using Ingenuity Pathway Analysis, Ingenuity® Systems (Green = expression decreased, red = expression increased in 4X relative to 2X udder halves on day 21 of lactation).

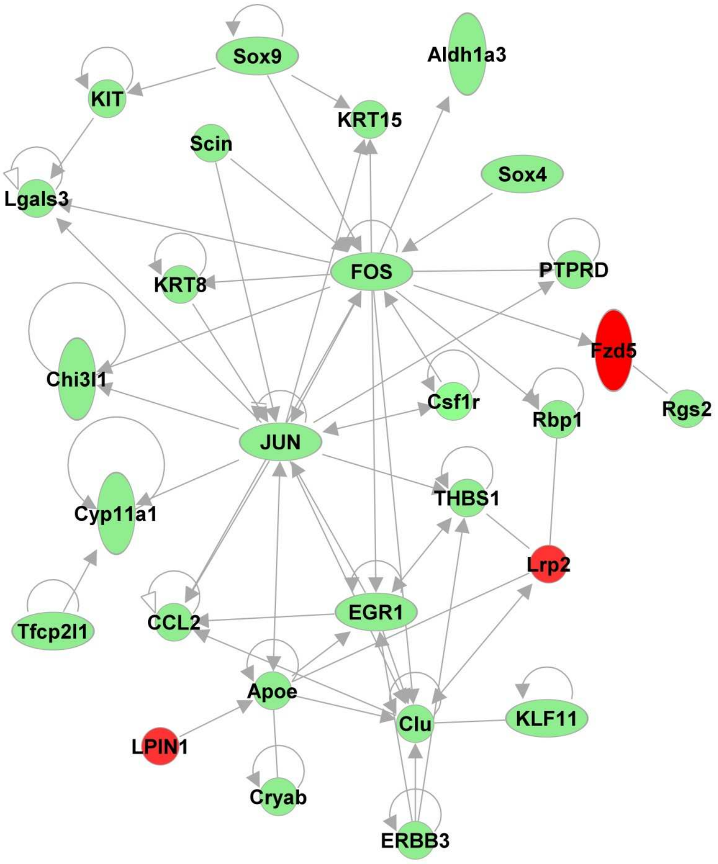

Supplement: Additional file 1: Figure S1 — Known potential connections between genes identified by a significant treatment by time interaction, characterized by changes in differential gene expression between 2× and 4× udder halves over time. Figure generated using Ingenuity Pathway Analysis, Ingenuity® Systems (Green = expression decreased, red = expression increased in 4× relative to 2× udder halves on day 21 of lactation). [file 1471-2164-14-296-S1.pdf]
