# Supplementary material for: Milk yield responses to changes in milking frequency during early lactation are associated with coordinated and persistent changes in mammary gene expression
Source: BMC Genomics. 2013 May 2;14:296. doi: 10.1186/1471-2164-14-296 (PMC3658990; doi:10.1186/1471-2164-14-296)
Supplement: Additional file 2: Figure S2 — Expression of A. chitinase 3-like (CHI3L)-1, B. clusterin, C. early growth response (EGR)-1, D. sex determining region Y-box (SOX)-4, and E. low density lipoprotein related protein (LRP)-2 mRNA in mammary tissue of cows assigned to unilateral frequent milking (twice daily milking (2×) of the left udder half, four times daily milking (4×) of the right udder half) during d 1 to 21 of lactation. Mammary biopsies from both udder halves were obtained on d 21, 23, and 40 of lactation. Each bar represents Least Squares Mean ± pooled standard error mRNA expression normalized to β-2 microglobulin. Line graph (secondary x-axis) represents least squares mean difference (4× minus 2×) ± pooled SE mRNA expression normalized to β-2 microglobulin. Solid horizontal line represents zero on the secondary x-axis, indicating no differential expression. Expression of CHI3L1, clusterin, EGR1, and SOX4 was decreased in 4× udder halves at 21 days in milk (P < 0.07), whereas expression of LRP2 was increased in 4× udder halves (P < 0.01). Expression of EGR1 was increased in 4× udder halves at 23 days in milk (P < 0.10), whereas expression of all other genes was similar across udder halves (P > 0.20). Expression of clusterin, EGR1, and SOX4 was decreased in 4× udder halves at 40 days in milk (P < 0.07), whereas expression of all other genes was similar across udder halves (P > 0.20). Differential expression of all genes changed over time (P < 0.07). [file 1471-2164-14-296-S2.pdf]

Supplemental Figure 2. Expression of **A.** chitinase 3-like (**CHI3L**)-1, **B.** clusterin, **C.** early growth response (**EGR**)-1, **D.** sex determining region Y-box (**SOX**)-4, and **E.** low density lipoprotein related protein (**LRP**)-2 mRNA in mammary tissue of cows assigned to unilateral frequent milking (twice daily milking (**2X**) of the left udder half, four times daily milking (**4X**) of the right udder half) during d 1 to 21 of lactation. Mammary biopsies from both udder halves were obtained on d 21, 23, and 40 of lactation. Each bar represents Least Squares Mean ± pooled standard error mRNA expression normalized to β-2 microglobulin. Line graph (secondary x-axis) represents least squares mean difference (4X minus 2X) ± pooled SE mRNA expression normalized to β-2 microglobulin. Solid horizontal line represents zero on the secondary axis x-axis, indicating no differential expression. Expression of *CHI3L1*, clusterin, *EGR1*, and *SOX4* was decreased in 4X udder halves at 21 days in milk ($P < 0.07$), whereas expression of *LRP2* was increased in 4X udder halves ($P < 0.01$). Expression of *EGR1* was increased in 4X udder halves at 23 days in milk ($P < 0.10$), whereas expression of all other genes was similar across udder halves ($P > 0.20$). Expression of clusterin, *EGR1*, and *SOX4* was decreased in 4X udder halves at 40 days in milk ($P < 0.07$), whereas expression of all other genes was similar across udder halves ($P > 0.20$). Differential expression of all genes changed over time ($P < 0.07$).

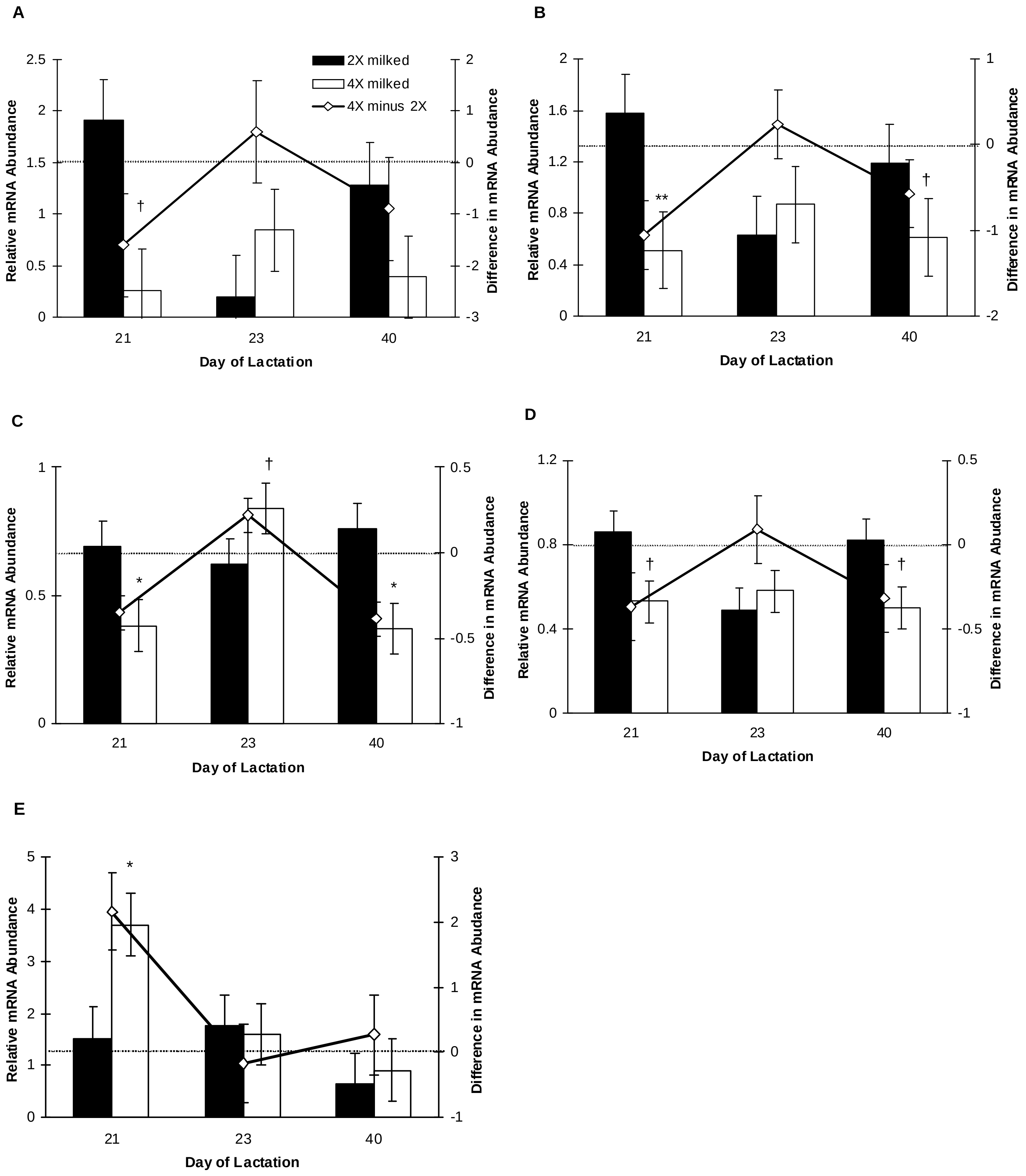
A
B
C
D
E
2X milked
4X milked
4X minus 2X
Relative mRNA Abundance
Difference in mRNA Abundance
Difference in mRNA Abudance
Day of Lactation
21
23
40
†
**
*
