# Supplementary material for: Milk yield responses to changes in milking frequency during early lactation are associated with coordinated and persistent changes in mammary gene expression
Source: BMC Genomics. 2013 May 2;14:296. doi: 10.1186/1471-2164-14-296 (PMC3658990; doi:10.1186/1471-2164-14-296)
Supplement: Additional file 3: Table S1 — Predicted activation state of growth factors and cytokines based on differential gene expression induced by IMF1. [file 1471-2164-14-296-S3.docx]

**Supplemental Table 1.** Predicted activation state of growth factors and cytokines based on differential gene expression induced by IMF^1^.

| **Effect** | **Regulator** | **Molecule Type** | **Predicted State^3^** | **z-score** | **p-value** | **Target Molecules In Dataset** |
| --- | --- | --- | --- | --- | --- | --- |
| IMF d21 | BDNF | growth factor | Inhibited | -2.0 | 9.1E-04 | Aldh7a1,Casp3,Cav2,Cdkn1c,Dnajb5,Egr1,Fos,Glul,Hivep1,Hsd17b12,Hspa5,Itpr1,Jun,Klf10,  Mapt,Ryr2,S100a10,Sorl1,Tiam1,Tiparp |
| IMF d23 | BDNF | growth factor | NS | NS | NS | None |
| IMF d40 | BDNF | growth factor | NS | NS | NS | None |
| IMF*Time^2^ | BDNF | growth factor | n/a | n/a | 9.0E-03 | Adrb2,Cdkn1c,Dnajb5,Egr1,Fos,Hivep1,Jun,Klf10,Mapt,P4ha1,S100a10 |
| IMF d21 | C5 | cytokine | Inhibited | -2.2 | 1.9E-01 | Ccl2,Cybb,Egr1,F3,Hivep1,Icam1,Itgb2,Slc25a15 |
| IMF d23 | C5 | cytokine | NS | NS | NS | None |
| IMF d40 | C5 | cytokine | NS | NS | NS | None |
| IMF*Time | C5 | cytokine | n/a | n/a | 7.9E-03 | Apcs,Ccl2,Cybb,Egr1,Hivep1,Met,Sf1,Slc25a15 |
| IMF d21 | EGF | growth factor | Inhibited | -2.6 | 1.0E-05 | B4galt5,Casp3,Cd44,Cldn7,Clu,Csf1r,Ctgf,Cyp11a1,Egr1,Eif2ak3,Elf3,Epas1,Erbb2,Errfi1,Ets2,Fos,Gak,Gata2,Gfpt1,Grb7,Icam1,Ier3,Igfbp3,Irs1,Itga2,Jun,Junb,Klf10,Krt15,Limk2,Ltf,Mcl1,Pdcd4,Pfkm,Ppard,Prkch,Pthlh,S100a10,S100a11,Serpina1,Spi1,Tgfbr2,Tgif1,Thbs1,Timp1,Tnfaip1,Tnfrsf12a,Zfp36l2 |
| IMF d23 | EGF | growth factor | NS | NS | NS | None |
| IMF d40 | EGF | growth factor | NS | NS | NS | None |
| IMF*Time | EGF | growth factor | n/a | n/a | 4.9E-05 | Afp,B4galt5,Clu,Cyp11a1,Egr1,Eif2ak3,Fos,Gata2,Gfpt1,Grb7,Gsk3b,Ier3,Igfbp3,Itga2,Jun,Junb,Klf10,Limk2,Ltf,Mcl1,Pdcd4,Ppard,Prkch,Ptges,S100a10,S100a11,Snai2,Thbs1,Zfp36l2 |
| IMF d21 | GH | growth factor | NS | NS | NS | None |
| IMF d23 | GH | growth factor | NS | NS | NS | None |
| IMF d40 | GH | growth factor | NS | NS | NS | None |
| IMF*Time | GH | growth factor | n/a | n/a | 9.5E-03 | Atf4,Clu,Cyb5b,Egr1,Erbb3,Fos,Gata2,Igfbp3,Jun,Junb,Lipe,Mgst1,Rfc1 |
| IMF d21 | IFNG | cytokine | Inhibited | -3.1 | 5.9E-07 | Ablim3,Aldh7a1,Arfgap3,Arg2,Atf4,Btg1,C1qc,Capn3,Casp3,Ccl2,Ccr5,Cd14,Cd200,Cd276,Cd36,Cd44,Cfb,Clip2,Csf1r,Ctgf,Ctsb,Ctsc,Cyb561,Cybb,Cyp11a1,Cyp27a1,Ddb2,Egr1,Emid1,Erap2,Erbb2,Ets2,F3,Fabp5,Fhl2,Flrt2,Fos,Gprc5b,Icam1,Ier3,Ifi30,Ifih1,Ifit5,Irak1,Isg20,Itgb2,Jun,Junb,Kdr,Klf10,Klf6,Kmo,Krt15,Lgals3,Map3k8,Marcksl1,Mrc1,Myd88,Ppard,Prkca,Prkcd,Psmb10,Pthlh,Ptpn1,Rac2,Rarres1,Rfxank,Rorc,S100a10,Saa2,Serp1,Serpina1,Sirpa,Slc2a1,Slc40a1,Spi1,Tgfb2,Tgif1,Thbs1,Timp1,Timp4,Tlr2,Tlr3,Tnfrsf12a,Trim8,Tyrobp,Ube2e1,Ube2l6,Vsnl1 |
| IMF d23 | IFNG | cytokine | NS | NS | NS | None |
| IMF d40 | IFNG | cytokine | NS | NS | NS | None |
| IMF*Time | IFNG | cytokine | n/a | n/a | 3.8E-03 | Ablim3,Atf4,Capn3,Ccl2,Cfb,Ctsb,Ctsc,Cyb561,Cybb,Cyp11a1,Ddb2,Egr1,Erap2,Fbln1,Flrt2,  Fos,Hspb1,Ier3,Isg20,Itgav,Jun,Junb,Klf10,Klf6,Lgals3,Map2k2,Map3k8,Marcksl1,Parp9,Ppard,Prdm1,Prkca,Psmb10,Ptges,S100a10,Saa2,Sirpa,Slc40a1,Snai2,Thbs1,Tlr3,Vldlr |
| IMF d21 | IGF1 | growth factor | Inhibited | -2.3 | 8.5E-02 | Casp3,Cd44,Clu,Ctgf,Cyp11a1,Egr1,Epas1,F3,Fabp4,Fos,Gapdh,Gata2,Hspa5,Icam1,Ier3,Igfbp3,Irs1,Jun,Junb,Mapt,Mcl1,Nefm,Pdlim2,Slc2a1,Thbs1,Tnfrsf12a,Zfp36l1 |
| IMF d23 | IGF1 | growth factor | NS | NS | NS | None |
| IMF d40 | IGF1 | growth factor |  | 0.33 | 1.0E-02 | Anpep,Birc3,Ccnd2,Col2a1,Csf1,Ctnnb1,Egf,Egr1,Eln,Fbxo32,Fosb,Gh,Ghr,Grm7,Igf1,Igf1r,  Igfbp4,Il4r,Insr,Irs1,Junb,Mafg,Mmp14,Nox4,Prl,Srd5a1,Tg,Tjp1,Tshr,Zfp36l1 |
| IMF*Time | IGF1 | growth factor | n/a | n/a | 1.1E-01 | Clu,Cyp11a1,Egr1,Fos,Gata2,Ier3,Igfbp3,Itgav,Jun,Junb,Mapt,Mcl1,Pdlim2,Snai2,Thbs1 |
| IMF d21 | LEP | growth factor | Inhibited | -2.4 | 7.9E-02 | Acsl5,Ccl2,Cd14,Cd36,Cpt1a,Egr1,F3,Fabp4,Fmo5,Fos,Gpx1,Hspa5,Icam1,Igfbp3,Irs1,Jun,Junb,Mmp7,Nppc,Prkaa2,Saa2,Slc16a1,Sod1,Thbs1,Timp1,Tmed9,Ugp2 |
| IMF d23 | LEP | growth factor | NS | NS | NS | None |
| IMF d40 | LEP | growth factor | NS | NS | NS | None |
| **Effect** | **Regulator** | **Molecule Type** | **Predicted State** | **z-score** | **p-value** | **Target Molecules In Dataset** |
| IMF*Time | LEP | growth factor | n/a | n/a | 1.7E-01 | Acsl5,Asah1,Ccl2,Egr1,Fabp3,Fos,Gsk3b,Igfbp3,Jun,Junb,Lipe,Pemt,Saa2,Thbs1 |
| IMF d21 | PDGF BB | growth factor | Inhibited | -4.4 | 8.8E-06 | Arf5,Arg2,Cav2,Ccl2,Cd44,Cryab,Csf1r,Ctgf,Egr1,Enpp1,Ets2,F3,Fhl2,Fos,Fzd1,Ier3,Jun,Junb,Klf10,Klf6,Lgals3,Lmna,Map2,Mcl1,Mgst1,Myh1,Nfil3,Ppard,Rbp1,Rnd3,Slc1a1,Sphk1,Spi1,Taf9,Thbs1,Timp1,Tnfrsf12a,Trib1,Zfp36l1 |
| IMF d23 | PDGF BB | growth factor | NS | NS | NS | None |
| IMF d40 | PDGF BB | growth factor | NS | NS | NS | None |
| IMF*Time | PDGF BB | growth factor | n/a | n/a | 4.49E-06 | Adrb2,Ccl2,Cdo1,Cryab,Egr1,Enpp1,Fos,Fzd1,Ier3,Jun,Junb,Klf10,Klf6,Lgals3,Mcl1,Mgst1,  Ppard,Rbp1,Rfc5,Rnd3,Rpn2,Slc1a1,Sphk1,Thbs1,Trib3,Uck1 |
| IMF d21 | PRL | cytokine | Inhibited | -2.0 | 1.08E-03 | Akr1c3,Anxa2,Anxa5,Bag1,Bok,Clu,Ctsa,Ctsb,Cyp11a1,Egr1,Erbb2,Erbb3,Fos,Gna12,Igfbp3,  Jun,Krt15,Mgp,Mgst1,Parm1,Pdia4,Pdlim4,Sod1,Timp1,Timp2 |
| IMF d23 | PRL | cytokine | NS | NS | NS | None |
| IMF d40 | PRL | cytokine | NS | NS | NS | None |
| IMF*Time | PRL | cytokine | n/a | n/a | 2.95E-03 | Anxa2,Anxa3,Bok,Clu,Cpd,Ctsb,Cyp11a1,Egr1,Erbb3,Fos,Igfbp3,Jun,Mgp,Mgst1,Pdlim4 |
| IMF d21 | TGFB1 | growth factor | Inhibited | -2.4 | 6.48E-06 | Ankh,Anxa2,Aqp1,Atf4,Atg5,Atp13a3,Btg1,C1qc,C1s,Casp3,Cav2,Ccl2,Ccr5,Cd14,Cd36,Cd44,Cd46,Cdkn1c,Chi3l1,Cldn4,Clu,Cnn2,Col16a1,Cotl1,Csf1r,Ctgf,Ctps1,Ctsb,Ctsc,Cyb561,Cybb,Cyp11a1,Ddb2,Dock2,Egr1,Elf3,Enpp1,Ercc5,Espl1,F3,Fabp5,Fcer1a,Fos,Galm,Gatm,Ggt6,  Gnas,Hspa5,Icam1,Ier3,Ifi30,Ifih1,Ifrd1,Igfbp3,Irs1,Itga2,Itgb2,Itpr1,Jun,Junb,Kdelr2,Kdr,Klf10,Krt18,Krt7,Krt8,Lgals3,Limk2,Litaf,Lta4h,Ltbp1,Maoa,Mgp,Mid1,Mmp7,Mrc1,Ms4a8b,Mstn,Myd88,Myl6,Nt5e,Pa2g4,Pdhb,Pdlim4,Pnp,Ppard,Prkca,Prss22,Pthlh,Ptprk,Rbms1,Rgcc,Rorc,  Rybp,S100a10,S100a11,Saa2,Sar1a,Serp1,Serpina1,Serpinb2,Sirpa,Slc16a3,Slc2a1,Sphk1,Spi1,Sri,St3gal5,Tax1bp3,Tgfb2,Tgfbr2,Tgif1,Thbs1,Timp1,Timp2,Tlr2,Tmem184b,Tnfrsf12a,Trim2,Zfp36l2 |
| IMF d23 | TGFB1 | growth factor | NS | NS | NS | None |
| IMF d40 | TGFB1 | growth factor | NS | NS | NS | None |
| IMF*Time | TGFB1 | growth factor | n/a | n/a | 2.6E-05 | Afp,Ankh,Anxa2,Atf4,C1s,Cap1,Ccl2,Cdkn1c,Chi3l1,Cldn4,Clu,Cnn2,Ctsb,Ctsc,Cxcr6,Cyb561,Cybb,Cyp11a1,Ddb2,Dock2,Egr1,Eng,Enpp1,Ercc5,Fbln1,Fos,Galm,Ggt6,Gsn,Ier3,Igfbp3,Itga2,Itgav,Jun,Junb,Klf10,Krt7,Krt8,Lgals3,Limk2,Lta4h,Met,Mgp,Ms4a8b,Mstn,P4ha1,Pdhb,  Pdlim4,Pdzk1ip1,Ppard,Prkca,Rfc5,Rora,Rpn2,Rybp,S100a10,S100a11,Saa2,Sirpa,Slc35a1,  Snai2,Sphk1,Sri,Srm,St3gal5,Tax1bp3,Thbs1,Tyms,Zfp36l2 |

^1^IMF = increased milking frequency. Cows were assigned to twice daily milking of the left udder half (**2X**) and four times daily milking of the right udder half (**4X**) on d 1 to 21 of lactation followed by 2X thereafter. Mammary biopsies were obtained on days (**d**) 21, 23, and 40 of lactation. Differential gene expression was detected using Affymetrix GeneChip^®^ Bovine Genome Array and for pathway analysis, genes were considered differentially expressed when the *P*-value was < 0.01. Pathway analysis was conducted using Ingenuity Pathway Analysis software (Ingenuity^®^ Systems, [www.ingenuity.com](http://www.ingenuity.com)). Only the molecules for which there was significant enrichment (*P* < 0.05 at one or more time points) are shown.

^2^IMF*Time = change in differential gene expression between 2X and 4X udder halves on day 21 vs. that on day 23

^3^Predicted state = predicted activation state in 4X vs. 2X udder halves
