# Supplementary material for: Milk yield responses to changes in milking frequency during early lactation are associated with coordinated and persistent changes in mammary gene expression
Source: BMC Genomics. 2013 May 2;14:296. doi: 10.1186/1471-2164-14-296 (PMC3658990; doi:10.1186/1471-2164-14-296)
Supplement: Additional file 4: Table S2 — Enrichment of canonical pathways based on genes differentially expressed in response to IMF1. [file 1471-2164-14-296-S4.docx]

**Supplemental Table 2.** Enrichment of canonical pathways based on genes differentially expressed in response to IMF^1^.

| **Ingenuity Canonical Pathways** | **Effect** | **p-value** | **Molecules** |
| --- | --- | --- | --- |
| Androgen Signaling | IMF d21 | NS | None |
|  | IMF d23 | NS | None |
|  | IMF d40 | 4.8E-02 | Prkacb,Gnas,Gna15,Prkcd,Crebbp,Gnb5,Ncoa1,Gnai1,Prkd3,Gng7,Prkca,Prkcg |
|  | IMF*Time^2^ | NS | None |
| Axonal Guidance Signaling | IMF d21 | 1.7E-02 | Rac2,Erap2,Mmp7,Myl6,Limk2,Fzd1,Tubb2b,Pak1,Gli3,Sdc2,Ablim3,Smo,Psmd14,Erbb2,Rassf5,Sema4a,Prkca,  Gnas,Arhgef15,Gna12,Itga2,Ppp3cc,Efna4,Gng5,Efna1,Rock1,Dock1,Prkar2b,Glis2,Prkcd,Prkch,Gng2,Fzd7 |
|  | IMF d23 | NS | None |
|  | IMF d40 | 3.3E-03 | Prkacb,Bmp15,Klc1,Unc5a,Gnb5,Abl1,Egf,Ablim1,Gng7,Wasl,Mysm1,Igf1,Gna15,Myl4,Erbb2,Pik3r2,Prkd3,  Prkca,Pik3c2b,Arhgef12,Gnas,Adam15,C9orf3,Ptch1,Gnai1,Pdgfb,Prkcg,Efna1,Rock1,Dock1,Ptpn11,Glis2,Prkcd,Adam9 |
|  | IMF*Time | NS | None |
| Calcium Signaling | IMF d21 | NS | None |
|  | IMF d23 | NS | None |
|  | IMF d40 | 3.3E-02 | Prkacb,Atp2b1,Tnnc1,Crebbp,Ryr2,Chrnb4,Myh7b,Trpc3,Grina,Hdac6,Camk2d,Hdac7,Myl4,Asph,Slc8a1 |
|  | IMF*Time | NS | None |
| CCR5 Signaling in Macrophages | IMF d21 | 1.1E-02 | Fos,Ccr5,Gnas,Jun,Prkcd,Prkch,Gng2,Mapk12,Gng5,Prkca |
|  | IMF d23 | NS | None |
|  | IMF d40 | 1.9E-02 | Gnas,Prkcd,Gnb5,Mapk10,Gnai1,Prkd3,Gng7,Prkca,Prkcg |
|  | IMF*Time | 9.5E-03 | Cd3g,Fos,Jun,Prkch,Mapk12,Gng5,Prkca |
| Complement System | IMF d21 | 1.1E-02 | C1s,Cfb,Cfi,C1qc,Cd46,Cfh |
|  | IMF d23 | NS | None |
|  | IMF d40 | NS | None |
|  | IMF*Time | 3.5E-03 | C4bpa,C1s,Cfb,Cfi,Cfh |
| CRH Signaling | IMF d21 | 5.8E-03 | Gnas,Itpr1,Mapk12,Atf2,Braf,Fos,Jun,Prkar2b,Gli3,Prkcd,Smo,Atf4,Prkch,Prkca |
|  | IMF d23 | NS | None |
|  | IMF d40 | NS | None |
|  | IMF*Time | 2.7E-02 | Fos,Jun,Gli3,Map2k2,Atf4,Prkch,Mapk12,Prkca |
| CXCR4 Signaling | IMF d21 | 4.3E-03 | Gnas,Myl6,Gna12,Egr1,Itpr1,Mapk12,Gng5,Rock1,Fos,Dock1,Pak1,Jun,Rhoq,Rnd3,Prkcd,Rhou,Prkch,Gng2,Prkca |
|  | IMF d23 | NS | None |
|  | IMF d40 | 9.8E-03 | Pik3c2b,Gnas,Egr1,Gnb5,Gnai1,Gng7,Prkcg,Rock1,Dock1,Gna15,Prkcd,Mapk10,Myl4,Pik3r2,Rhof,Prkd3,Prkca |
|  | IMF*Time | NS | None |
| Ephrin B Signaling | IMF d21 | NS | None |
|  | IMF d23 | NS | None |
|  | IMF d40 | 2.4E-02 | Rock1,Vav2,Gnas,Gna15,Gnb5,Gnai1,Ctnnb1,Gng7,Hnrnpk |
|  | IMF*Time | NS | None |
| Ephrin Receptor Signaling | IMF d21 | 3.2E-02 | Rac2,Gnas,Arhgef15,Gna12,Itga2,Limk2,Gng5,Efna4,Atf2,Efna1,Fgf1,Rock1,Pak1,Sdc2,Atf4,Dok1,Gng2 |
|  | IMF d23 | NS | None |
|  | IMF d40 | NS | None |
|  | IMF*Time | NS | None |
| GNRH Signaling | IMF d21 | 6.3E-03 | Gnas,Map3k6,Egr1,Itpr1,Mapk12,Atf2,Fos,Pak1,Camk2d,Jun,Prkar2b,Prkcd,Atf4,Map3k8,Prkch,Prkca |
|  | IMF d23 | NS | None |
|  | IMF d40 | 1.7E-02 | Prkacb,Gnas,Egr1,Crebbp,Gnai1,Egf,Prkcg,Map3k12,Camk2d,Gna15,Prkcd,Mapk10,Prkd3,Prkca |
|  | IMF*Time | 3.2E-02 | Fos,Jun,Map2k2,Egr1,Atf4,Map3k8,Prkch,Mapk12,Prkca |
| **Ingenuity Canonical Pathways** | **Effect** | **p-value** | **Molecules** |
| Growth Hormone Signaling | IMF d21 | NS | None |
|  | IMF d23 | NS | None |
|  | IMF d40 | 8.3E-04 | Rps6kb1,Pik3c2b,Ghr,Igf1,Prkcd,Irs1,Igf1r,Pik3r2,Prkd3,Gh,Prkca,Prkcg |
|  | IMF*Time | NS | None |
| HGF Signaling | IMF d21 | 1.3E-02 | Map3k6,Ets2,Mapk12,Atf2,Dock1,Fos,Pak1,Elf3,Jun,Prkcd,Map3k8,Prkch,Prkca |
|  | IMF d23 | NS | None |
|  | IMF d40 | 3.9E-02 | Dock1,Map3k12,Pik3c2b,Ptpn11,Prkcd,Mapk10,Pik3r2,Prkd3,Elf1,Prkca,Prkcg |
|  | IMF*Time | 2.5E-02 | Met,Fos,Jun,Map2k2,Map3k8,Prkch,Mapk12,Prkca |
| IGF-1 Signaling | IMF d21 | NS | None |
|  | IMF d23 | NS | None |
|  | IMF d40 | 4.9E-02 | Prkacb,Rps6kb1,Igfbp4,Pik3c2b,Igf1,Ptpn11,Irs1,Igf1r,Csnk2b,Pik3r2,Grb10 |
|  | IMF*Time | NS | None |
| IL-8 Signaling | IMF d21 | 2.0E-03 | Rac2,Icam1,Gnas,Gna12,Limk2,Mapk12,Cstb,Gng5,Irak1,Braf,Rock1,Fos,Itgb2,Jun,Rhoq,Rnd3,Prkcd,Kdr,Cybb,  Rhou,Prkch,Gng2,Prkca |
|  | IMF d23 | NS | None |
|  | IMF d40 | 3.9E-03 | Pik3c2b,Rps6kb1,Nox4,Gnas,Flt4,Gnai1,Gnb5,Egf,Gng7,Azu1,Prkcg,Traf6,Rock1,Ccnd2,Prkcd,Ncf2,Mapk10,  Pik3r2,Rhof,Prkd3,Prkca |
|  | IMF*Time | 1.4E-02 | Limk2,Mapk12,Iqgap1,Gng5,Cstb,Fos,Jun,Map2k2,Rnd3,Itgav,Cybb,Prkch,Prkca |
| Leukocyte Extravasation Signaling | IMF d21 | 7.2E-03 | Rac2,Mmp7,Icam1,Myl6,Itga2,Cldn7,Mapk12,Rock1,Itgb2,Timp4,Mmp23b,Cldn4,Timp1,Prkcd,Fer,Cd44,Cybb,  Prkch,Rassf5,Prkca,Timp2 |
|  | IMF d23 | NS | None |
|  | IMF d40 | 3.0E-03 | Vav2,Pik3c2b,Mmp16,Mmp14,Cldn18,Abl1,Gnai1,Prkcg,Rock1,Wasl,Ptpn11,Prkcd,Ncf2,Mapk10,Pik3r2,Actn4,  Ctnnb1,Prkd3,Mmp17,Actn1,Prkca |
|  | IMF*Time | NS | None |
| NRF2-mediated Oxidative Stress Response | IMF d21 | 3.9E-02 | Mgst1,Sod1,Gstm3,Hspb8,Slc35a2,Junb,Dnajb9,Fos,Gstt1,Jun,Prkcd,Atf4,Prkch,Dnajb1,Eif2ak3,Dnajb5,Prkca,  Gstk1 |
|  | IMF d23 | NS | None |
|  | IMF d40 | NS | None |
|  | IMF*Time | 3.9E-03 | Mgst1,Gstm3,Junb,Gstt1,Fos,Jun,Map2k2,Atf4,Prkch,Gsk3b,Eif2ak3,Dnajb5,Ephx1,Prkca |
| Production of NO and ROS in Macrophages | IMF d21 | 1.0E-02 | Map3k6,Clu,Arg2,Mapk12,Spi1,Tlr2,Fos,Lyz,Ppp1r3d,Rhoq,Jun,Rnd3,Prkcd,Cybb,Rhou,Map3k8,Serpina1,Prkch,Sirpa,Prkca |
|  | IMF d23 | NS | None |
|  | IMF d40 | NS | None |
|  | IMF*Time | 4.7E-02 | Fos,Jun,Ppp1r10,Rnd3,Cybb,Clu,Map3k8,Prkch,Mapk12,Sirpa,Prkca |
| Prolactin Signaling | IMF d21 | NS | None |
|  | IMF d23 | NS | None |
|  | IMF d40 | 2.1E-02 | Pik3c2b,Ptpn11,Prl,Prkcd,Irs1,Crebbp,Pik3r2,Prkd3,Prkca,Prkcg |
|  | IMF*Time | NS | None |

^1^IMF = increased milking frequency. Cows were assigned to twice daily milking of the left udder half (**2X**) and four times daily milking of the right udder half (**4X**) on d 1 to 21 of lactation followed by 2X thereafter. Mammary biopsies were obtained on days (**d**) 21, 23, and 40 of lactation. Differential gene expression was detected using Affymetrix GeneChip^®^ Bovine Genome Array and for pathway analysis, genes were considered differentially expressed when the *P*-value was < 0.01. Pathway analysis was conducted using Ingenuity Pathway Analysis software (Ingenuity^®^ Systems, [www.ingenuity.com](http://www.ingenuity.com)). Only the pathways for which there was significant enrichment (*P* < 0.05 at one or more time points) are shown.

^2^IMF*Time = change in differential gene expression between 2X and 4X udder halves on day 21 vs. that on day 23.
